# Rab35 protein regulates evoked exocytosis of endothelial Weibel–Palade bodies

**DOI:** 10.1074/jbc.M116.773333

**Published:** 2017-05-31

**Authors:** Anja Biesemann, Alexandra Gorontzi, Francis Barr, Volker Gerke

**Affiliations:** From the ‡ Institute of Medical Biochemistry, Center for Molecular Biology of Inflammation, University of Münster, D-48149 Münster, Germany and; the § Department of Biochemistry, University of Oxford, Oxford OX1 3QU, United Kingdom

**Keywords:** calcium, endothelial cell, membrane trafficking, Rab, vascular biology, Calcium, endothelial cells, membrane traffic, von-Willebrand factor

## Abstract

Weibel–Palade bodies (WPB) are secretory organelles of endothelial cells that undergo evoked exocytosis following intracellular Ca^2+^ or cAMP elevation, thereby supplying the vasculature with factors controlling hemostasis. Several cytosolic and membrane-associated proteins, including the Rab family members Rab3, Rab15, and Rab27a, have been implicated in regulating the acute exocytosis of WPB. Here, we carried out a genome-wide screen to identify Rab pathways affecting WPB exocytosis. Overexpression of a specific subset of Rab GTPase–activating proteins (RabGAPs) inhibited histamine-evoked, Ca^2+^-dependent WPB exocytosis, presumably by inactivating the target Rab GTPases. Among these RabGAPs, we concentrated on TBC1D10A and showed that the inhibitory effect depends on its GAP activity. We confirmed that Rab35 was a target Rab of TBC1D10A in human endothelial cells; Rab35 interacted with TBC1D10A, and expression of the GAP-insensitive Rab35(Q67A) mutant rescued the inhibitory effect of TBC1D10A overexpression on WPB exocytosis. Furthermore, knockdown of Rab35 and expression of a dominant-negative Rab35 mutant both inhibited histamine-evoked secretion of the WPB cargos von Willebrand factor and P-selectin. Pulldown and co-immunoprecipitation experiments identified the ArfGAP with coiled-coil, Ank repeat, and pleckstrin homology domain–containing protein ACAP2 as an Rab35 effector in endothelial cells, and depletion as well as overexpression approaches revealed that ACAP2 acts as a negative regulator of WPB exocytosis. Interestingly, a known ACAP2 target, the small GTPase Arf6, supported histamine-evoked WPB exocytosis, as shown by knockdown and overexpression of a dominant-negative Arf6 mutant. Our data identify Rab35 as a novel regulator of WPB exocytosis, most likely acting through the downstream effectors ACAP2 and Arf6.

## Introduction

Endothelial cells supply the vasculature with factors controlling coagulation (*e.g.* the multimeric glycoprotein von Willebrand factor (VWF)^3^) and the local recruitment of leukocytes (*e.g.* the adhesion receptor P-selectin). These factors are stored inside the cells in secretory granules, the Weibel–Palade bodies (WPB), which release their content following endothelial stimulation and intracellular Ca^2+^ or cAMP elevation. Thus, regulated exocytosis of WPB serves important functions in the control of vascular homeostasis (for reviews, see Refs. 1–4). WPB are considered lysosome-related organelles because some of their contents (*e.g.* the tetraspanin and P-selectin cofactor CD63) are delivered from endosomes to maturing WPB following their initial emergence at the TGN. Maturation of WPB is also characterized by the processing of VWF, which forms condensed tubules inside WPB and thereby determines the characteristic rodlike shape of these organelles (for reviews, see Refs. 5 and 6).

A number of factors participating in the maturation and evoked exocytosis of WPB have been described. These include the small GTPases RalA and Rap1 as well as their regulators RalGDS and Epac (7), the dynein–dynactin complex and the actin regulator RhoA (5, 8), members of the SNARE family (9, 10), the phospholipid-metabolizing enzyme phospholipase D1 (11), and several members of the annexin family (12). Importantly, different members of the Rab family of small GTPases have been shown to play key roles in regulating WPB maturation and exocytosis. The Rab3 isoforms 3b and 3d appear to function in regulating maturation and secretion (13, 14), and Rab15 has been shown to positively regulate WPB exocytosis, cooperating with another Rab GTPase, Rab27a (15). Rab27a seems to serve different functions in WPB exocytosis that are probably determined by various effectors. In conjunction with MyRIP and myosin Va, Rab27a mediates an anchorage of WPB at the cortical actin cytoskeleton, allowing complete maturation of VWF before exocytosis (16, 17). On the other hand, Rab27a has also been implicated in supporting WPB secretion by acting through the effector Slp4a (14). The three above-mentioned Rabs shown to function in WPB exocytosis, Rab3, Rab15, and Rab27a, also localize to WPB in endothelial cells. In addition, a comprehensive screen recording the subcellular localization of GFP-tagged Rab proteins (Rab1–43) in endothelial cells revealed a WPB localization of two other Rabs, Rab33a and Rab37. However, these two Rabs appeared not to be functionally involved in WPB exocytosis evoked by a mixture of ATP, VEGF, and basic fibroblast growth factor (15). Thus, several Rab proteins are likely to play distinct roles in WPB exocytosis, although their exact mode of action has not been delineated in most cases.

To obtain a comprehensive view on the functional involvement of different Rab proteins in WPB exocytosis, we performed a screen employing all RabGAPs encoded in the human genome as inhibitors of Rab activity. The RabGAPs were expressed transiently in endothelial cells, and their effect on histamine-evoked release of VWF was analyzed by employing a novel, flow cytometry–based secretion assay. This identified TBC1D10A as one of the RabGAPs inhibiting WPB exocytosis and Rab35 as both a target of TBC1D10A and a positive regulator of WPB exocytosis in HUVEC. Interaction studies revealed that ACAP2 could serve as a downstream effector of Rab35 in endothelial cells. ACAP2 can regulate Arf6, which had previously been implicated in VWF secretion induced by Shiga toxin (18), and we show here that Arf6 also affects histamine-evoked WPB exocytosis in a positive manner. Our data suggest a model whereby histamine stimulation of HUVEC activates Rab35 that supports WPB exocytosis through a downstream effector cascade involving ACAP2 and Arf6.

## Results

### A novel FACS-based assay identifies several RabGAPs as inhibitors of WPB exocytosis

**Figure 1.**
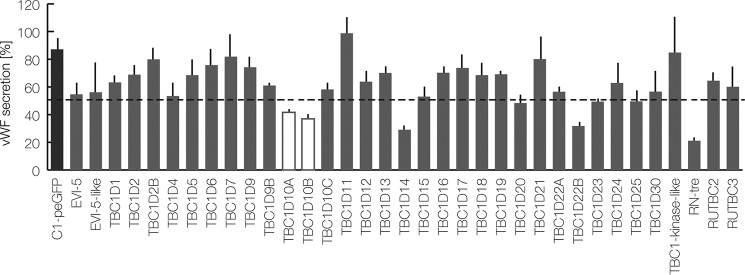
**A FACS-based RabGAP screen identifies TBC1D10A/B as regulators of Ca^2+^-mediated VWF secretion.** HUVEC expressing 35 different GFP-tagged RabGAPs or C1-pEGFP, which served as a reference, were stimulated with histamine, and the relative amount of acute VWF secretion was determined by a FACS-based antibody capture assay (see “Experimental procedures”). GAPs reducing the release of VWF by > 50% of the control were considered as regulators of WPB exocytosis. The mean of three independent experiments is shown in the graph. *Error bars*, S.E.

To record the effect of the different RabGAPs expressed in the human genome on histamine-evoked WPB exocytosis, we developed a novel high-throughput analysis. Therefore, primary human umbilical vein endothelial cells (HUVEC) were stimulated with histamine in the presence of extracellular anti-VWF antibodies, and the amount of antibody that was captured by exocytosed VWF was quantified by flow cytometry (supplemental Fig. S1). This antibody capture had previously been shown to represent an indirect but reliable measure of evoked WPB exocytosis (13). All individual RabGAPs were expressed transiently as GFP fusion proteins in HUVEC, and the effect of the respective RabGAP overexpression on anti-VWF antibody capture was quantified specifically in GFP-positive cells by FACS gating. Fig. 1 shows a summary of the results. It reveals that most RabGAPs, when overexpressed in HUVEC, have no significant stimulatory or inhibitory effect on the anti-VWF capture. However, overexpression of five of the GAPs, RN-tre, TBC1D14, TBC1D22B, TBC1D10A, and TBC1D10B, resulted in a > 50% reduction of antibody capture, indicative of an inhibitory effect on VWF secretion. It should be noted that the effect of the RabGAPs TBC1D3B, TBC1D8, RUTBC1, and USP6 could not be determined due to very low expression levels.

Among the inhibitory GAPs identified, we focused on TBC1D10A and TBC1D10B because they had been implicated in secretory events in other cell types but had not been analyzed in the context of endothelial cell secretion (19, 20). In a first set of experiments, we performed siRNA-mediated knockdown of these RabGAPs and determined the effect on WPB exocytosis by quantifying VWF release into the culture supernatant after histamine stimulation. Depletion of TBC1D10A but not TBC1D10B caused a slight but significant stimulatory effect on histamine-evoked VWF secretion (supplemental Fig. S2). This phenotype is in line with the inhibitory effect of TBC1D10A overexpression and further supports the notion that this GAP acts as a negative regulator of WPB exocytosis. Next, we recorded the intracellular localization of TBC1D10A and TBC1D10B, which were expressed as GFP-tagged derivatives in HUVEC. Both fusion proteins showed a general cytosolic plus plasma membrane localization with enrichment at intracellular and/or membrane-associated puncta. The puncta did not colocalize with WPB, as revealed by coexpression of the WPB marker VWF–RFP (supplemental Fig. S3).

**Figure 2.**
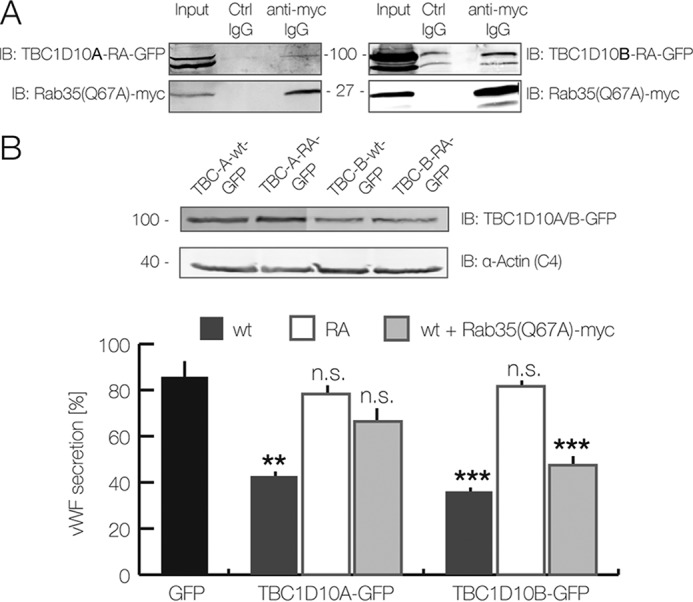
**TBC1D10A interacts with Rab35.** *A* and *B*, HEK-293T cells were co-transfected with constitutively active Rab35(Q67A)-Myc and catalytically inactive TBC1D10A(R160A)-GFP or TBC1D10B(R409A)-GFP, respectively. *A*, immunoblot showing the cell lysate as input and anti-Myc as well as isotype control-matched immunoprecipitates. 24 hpt, cells were treated with NaF/AlCl_3_ and lysed. Lysates were subjected to immunoprecipitation using anti-Myc antibodies. *IB*, immunoblot. *B*, Rab35(Q67A)-Myc rescues the inhibitory effect of TBC1D10A-GFP on VWF secretion. HUVEC were transfected with GFP-coupled WT TBC1D10A/B and their inactive mutants (RA). In some experiments, Rab35(Q67A)-Myc was co-transfected as indicated. The *top panel* shows an anti-GFP immunoblot of the respective cell lysates. Note that the GFP-coupled WT and mutant (RA) TBC1D10A/B constructs show similar expression levels. α-Actin was used as loading control. The *bottom panel* shows the results of VWF secretion assays carried out with the accordingly transfected cells. 24 hpt, cells were stimulated with histamine, and acute VWF secretion was determined using the FACS-based assay. The mean of seven independent experiments is shown in the graph. *Error bars*, S.E. (**, *p* < 0.01; ***, *p* < 0.001; one-way ANOVA and Tukey test).

To analyze whether the GAP activity toward a target Rab protein is required for the inhibitory action of TBC1D10A and TBC1D10B on WPB exocytosis, we generated point mutants that showed impaired GAP activity due to replacement of a crucial arginine residue in the active site by alanine (RA mutants) (21). In contrast to wild-type TBC1D10A and -B, ectopic expression of the RA mutants of both GAPs did not inhibit the histamine-evoked secretion of VWF (Fig. 2*B*).

### Rab35 is a target of TBC1D10A and a novel positive regulator of WPB exocytosis in HUVEC

Previous biochemical analyses had revealed that TBC1D10A to -C show GAP activity toward Rab35 and thereby inactivate it (19, 22). Therefore, we next determined whether TBC1D10A and -B also interact with Rab35 in HUVEC. To stabilize potentially transient interactions, co-immunoprecipitations (co-IPs) were carried out with lysates from cells expressing the inactive RA mutants of TBC1D10A and -B and the Q67A mutant of Rab35, which exhibits greatly reduced GTP hydrolysis when stimulated with TBC1D10A (21, 23–25). Whereas TBC1D10A-RA co-precipitated with Myc-tagged Rab35(Q67A) to a level well above that observed with control IgGs, higher nonspecific background binding was obtained for TBC1D10B-RA, precluding any statement concerning a specific co-IP of TBC1D10B-RA with Rab35(Q67A) (Fig. 2*A*). Given the interaction of at least TBC1D10A with Rab35, we next assessed whether the inhibitory effect of TBC1D10A and/or TBC1D10B on evoked WPB exocytosis is due to an interaction with and thus inactivation of Rab35. Therefore, the active Rab35(Q76A) mutant was expressed in HUVEC overexpressing TBC1D10A or TBC1D10B, and the effect on histamine-evoked WPB exocytosis was determined by quantifying VWF secretion via flow cytometry. The results are depicted in Fig. 2*B*. Whereas Rab35(Q67A) did not alter the inhibition of VWF secretion observed in TBC1D10B-overexpressing cells, Rab35(Q67A) expression compensated the inhibitory effect of TBC1D10A. This suggests that TBC1D10A but not TBC1D10B regulates Rab35 in the course of WPB exocytosis.

**Figure 3.**
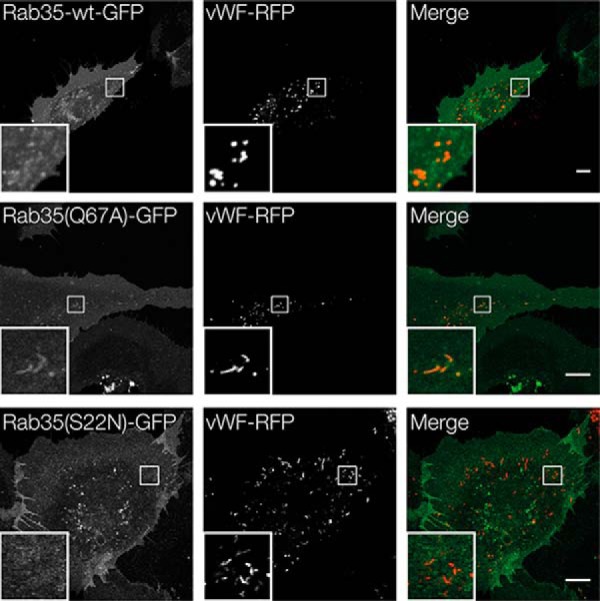
**Localization of GFP–Rab35 in HUVEC.** Cells were co-transfected with plasmids encoding VWF–RFP as a WPB marker and GFP-conjugated Rab35-WT, Rab35(Q67A), or Rab35(S22N). 24 hpt, fluorescence images were recorded of non-fixed cells using confocal microscopy. *Scale bar*, 10 μm.

**Figure 4.**
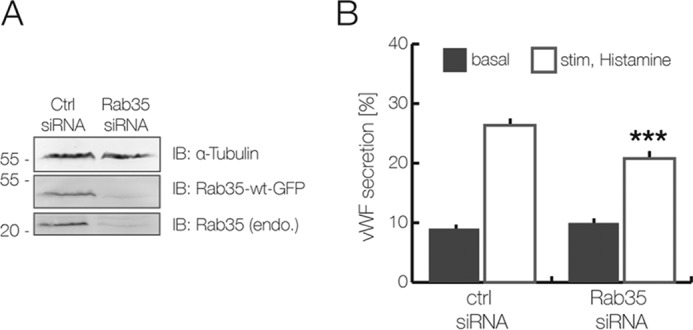
**Inhibitory effect of Rab35 knockdown on histamine-evoked VWF secretion.** *A* and *B*, HUVEC were transfected twice for 48 h with a Rab35-specific siRNA and a nonspecific siRNA, which served as negative control. *A*, Western blot analysis of Rab35-depleted cells, which were co-transfected with wild-type Rab35–GFP. The knockdown efficiency was determined by immunoblotting of total cell lysate using anti-GFP antibodies as well as anti-Rab35 antibodies to probe for the endogenous protein. α-Tubulin was used as loading control. *IB*, immunoblot. *B*, VWF secretion levels in Rab35-depleted cells. HUVEC were treated with histamine-containing stimulation medium and processed for ELISA-based secretion analysis as described under “Experimental procedures.” Results are expressed as the mean ± S.E. (*error bars*) of 12 independent experiments (***, *p* < 0.001; paired *t* test).

To further elucidate the role of Rab35 in regulated WPB exocytosis, we determined whether it can associate with WPB. Therefore, Rab35 wild type as well as its active (Q67A) and inactive (S22N) mutants were expressed as GFP-tagged versions, and their distribution was compared with that of VWF–RFP as a WPB marker. Rab35 showed a general cellular staining indicative of some plasma membrane association (Fig. 3) as well as an association with intracellular puncta, some of which representing VWF-labeled WPB. Interestingly, live cell experiments revealed that histamine stimulation induced a recruitment of some of the Rab35–GFP to WPB that fuse with the plasma membrane (supplemental Fig. S4). Rab35(Q67A) was also present in the cytosol and at the membrane and showed a partial colocalization with WPB. This colocalization was not observed for Rab35(S22N). Given the partial localization of Rab35 on WPB and the fact that Rab35(Q67A) can rescue the inhibition of VWF secretion triggered by overexpression of its GAP, TBC1D10A, we also assessed the consequences of Rab35 knockdown on VWF secretion. As expected from the above analyses, Rab35 depletion significantly interfered with histamine-evoked VWF secretion (Fig. 4). Thus, Rab35 is a positive regulator of WPB exocytosis and is most likely regulated by TBC1D10A in endothelial cells.

### ACAP2 is a Rab35 effector in endothelial cells

**Figure 5.**
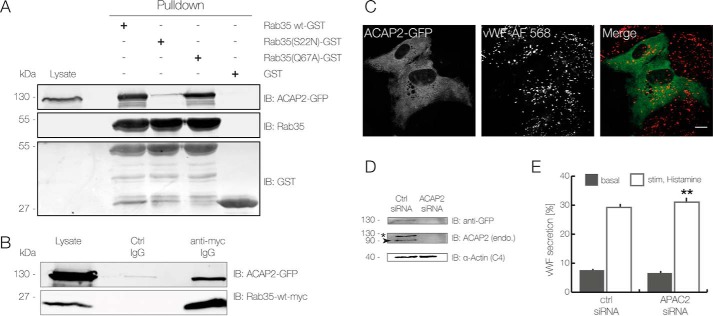
**ACAP2 is a potential Rab35 effector in HUVEC.** *A* and *B*, analysis of the interaction between Rab35 and ACAP2 using affinity chromatography (*A*) and immunoprecipitation (*B*). *A*, GST alone or the GST-coupled proteins Rab35-WT, Rab35(Q67A), and Rab35(S22N) were immobilized on GSH beads and incubated with lysates of HEK-293T cells overexpressing ACAP2–GFP. Bound proteins were released and analyzed by immunoblot using anti-GFP, anti-Rab35, and anti-GST antibodies. *IB*, immunoblot. *B*, lysates from HEK-293T cells expressing ACAP2–GFP and Rab35-WT–Myc were treated with anti-c-Myc antibodies or isotype-matched control antibodies and subjected to immunoprecipitation. Precipitated proteins and total lysates were analyzed by immunoblot using anti-GFP and anti-c-Myc antibodies to identify ACAP2 and Rab35, respectively. *C*, ACAP2 localization in HUVEC. Cells expressing ACAP2–GFP were fixed and processed for immunofluorescence using anti-VWF antibodies. Note that ACAP2–GFP shows a uniform distribution and no colocalization with WPB. *Scale bar*, 10 μm. *D* and *E*, siRNA-mediated ACAP2 knockdown in HUVEC. Cells were transfected twice for 48 h with an ACAP2 specific siRNA and a nonspecific control siRNA. *D*, immunoblot analysis of ACAP2-depleted cells, which were co-transfected with wild type ACAP2–GFP. The knockdown efficiency was determined by immunoblotting of total cell lysates using anti-GFP antibodies as well as an anti-ACAP2 antibody that recognizes the endogenous and the ectopically expressed GFP-coupled protein. α-Actin served as loading control. *, ACAP2–GFP; *arrow*, endogenous ACAP2. *E*, levels of VWF secretion in ACAP2-depleted cells as quantified by ELISA 48 hpt. HUVEC were treated with histamine-containing stimulation medium as described under “Experimental procedures.” Results are expressed as the mean ± S.E. (*error bars*) of 12 independent experiments (**, *p* < 0.01; paired *t* test).

Rab proteins function by regulating downstream effectors, typically in their activated, GTP-bound form. To identify such effectors in endothelial cells, which could potentially function in the regulation of WPB exocytosis, we subjected HUVEC lysates to affinity purification employing immobilized GST-tagged Rab35. Several proteins were found to specifically bind to Rab35-WT–GST and not to the GST control (supplemental Fig. S5). Eight of the bands enriched on the Rab35-WT–GST beads were subjected to identification by mass spectrometry, which revealed the presence of a known Rab35 effector, the coiled-coil, Ank repeat, and pleckstrin homology domain–containing protein ACAP2. An interaction between ACAP2 and wild-type Rab35 as well as Rab35(Q67A) in endothelial cells was confirmed by pulldown and co-IP experiments carried out with lysates of cells expressing GFP-tagged ACAP2 (Fig. 5, *A* and *B*). We also assessed the localization of ACAP2 in HUVEC by expressing ACAP2–GFP. This showed a rather homogeneous cytosolic distribution with no obvious localization at WPB, both in non-stimulated and in histamine-stimulated HUVEC (Fig. 5*C* and supplemental Fig. S6).

Next, we analyzed whether ACAP2 is also involved in WPB exocytosis. The protein was depleted by siRNA treatment (see Fig. 5*D* for knockdown efficiency), and the depleted cells were subjected to quantification of histamine-stimulated release of VWF. ACAP2 knockdown resulted in a minor but significant stimulatory effect on the evoked VWF secretion (Fig. 5*E*). Thus, it appears that ACAP2, in contrast to Rab35, is either not significantly involved in stimulated WPB exocytosis or acts as a moderate, negative regulator.

### The ACAP2 target Arf6 participates in evoked WPB exocytosis

**Figure 6.**
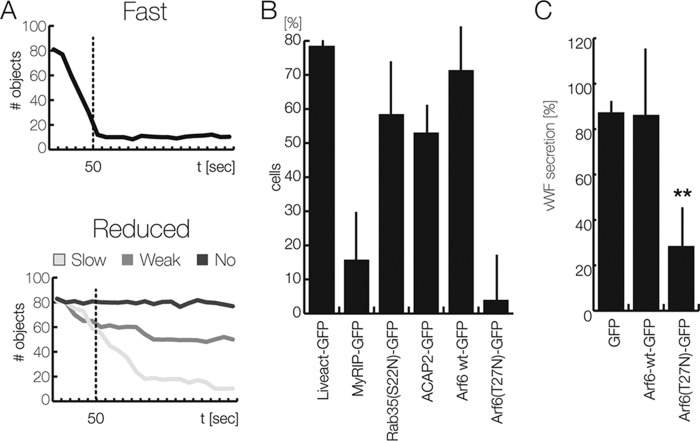
**Effect of Rab35 and different effectors on the kinetics of WPB exocytosis.** *A* and *B*, object disappearance measured by TIRF microscopy. HUVEC were co-transfected with Psellum–mRFP as a WPB marker and the different GFP-tagged constructs given in *B*; cultivated on collagen-coated, chambered slides; and subjected to TIRF imaging as described under “Experimental procedures.” Cells were stimulated with 100 μm histamine within the first 10 s of recording. *A*, the number of Psellum-mRFP–positive objects in the TIRF field was measured over time in individual cells, and the decrease in this number was taken as a measure of WPB exocytosis because this is accompanied by a release of Psellum–mRFP. A rapid decrease to low baseline levels of these numbers within 50 s was considered fast secretion (*top graph*). The majority of untreated or mock-treated cells (≥ 80%) showed this response. A minor fraction of untreated or mock-treated cells responded to histamine stimulation with a delayed (*light gray*), moderated (*gray*), or no (*dark gray*) reduction in the number of Psellum–mRFP objects. *B*, bar graph presenting the relative amount of HUVEC with fast WPB exocytosis as classified in *A*. Lifeact-GFP served as a positive reference, whereas MyRIP-GFP was considered a negative control (16). Results are expressed as mean ± S.E. (*error bars*) of 70 individual cells from 4–5 independent experiments. *C*, FACS-based VWF secretion assay. HUVEC transfected for 24 h with empty vector (which served as the 100% control), C1-pEGFP, Arf6-WT–GFP, or Arf6(T27N)–GFP were stimulated with histamine, and the relative amount of acute VWF secretion was determined by the FACS-based antibody capture assay (see “Experimental procedures” and supplemental Fig. S1). The mean of four independent experiments is shown in the graph. *Error bars*, S.E. (**, *p* < 0.01; one-way ANOVA and Tukey test).

Given the rather minor effect of ACAP2 knockdown on histamine-evoked VWF release, we also performed overexpression experiments to assess a potential role of the protein in WPB exocytosis. Because the transfection rate of our ACAP2 constructs in HUVEC was rather low (only 10–20% of transfected cells), we used a microscopy-based assay to record effects of ACAP2 overexpression on WPB exocytosis at the individual cell level. Therefore, HUVEC were transfected with an mRFP-tagged P-selectin derivative only containing the luminal/extracellular domain, herein referred to as Psellum. This construct is specifically targeted to WPB, which consequently will accumulate mRFP label in the transfected cells (16). Exocytosis of these WPB leads to a rapid release of the Psellum–mRFP construct and thus to a reduced number of mRFP-positive, intracellular objects (*i.e.* WPB that can be found at or close to the plasma membrane). The reduction of these peripheral Psellum-mRFP–positive WPB was visualized by total internal reflection fluorescence (TIRF) microscopy, which only illuminates objects within 100–200 nm of the coverslip–cell interface (*i.e.* the plasma membrane). TIRF microscopy recordings were analyzed by an object detection algorithm to quantify the number of mRFP-labeled WPB per cell over time. This analysis revealed that the majority of cells respond to histamine stimulation with a rapid decrease in mRFP-positive objects, most likely reflecting the exocytosis of Psellum-mRFP–containing WPB (Fig. 6*A*). Hence, using this assay, defects in evoked WPB exocytosis are reflected as a failure of histamine to elicit in the cell a decrease in the number of mRFP-positive objects or a slower kinetics of their decrease (Fig. 6*A*). Validity of the assay was first verified by expression of the widely established actin marker Lifeact-GFP as a negative control, showing no significant effect on the number of cells responding to histamine stimulation with a rapid disappearance of Psellum-mRFP–positive objects. On the other end, overexpression of the Rab27a effector MyRIP, which anchors maturing WPB at the actin cortex and thereby inhibits histamine-evoked WPB exocytosis (16, 26), induced a marked decrease in the rate of WPB disappearance after histamine stimulation, thus serving as a positive inhibitory control (Fig. 6*B*).

We next used the TIRF microscopy assay to determine overexpression phenotypes of the different proteins identified as effectors of WPB exocytosis in this study (Fig. 6*B*). Expression of Rab35(S22N)-GFP, the inactive mutant not localizing to WPB, slightly reduced the number of cells showing a rapid decrease of Psellum-mRFP–positive objects upon histamine stimulation and thus appeared to inhibit stimulated WPB exocytosis to some extent. Likewise, overexpression of ACAP2–GFP resulted in an inhibition, which is in line with the slight increase of histamine-evoked VWF secretion observed following ACAP2 knockdown (see above; Fig. 5*E*). Therefore, the combined results indicate that the Rab35 effector ACAP2 acts as a moderate negative regulator of Ca^2+^-dependent WPB exocytosis.

**Figure 7.**
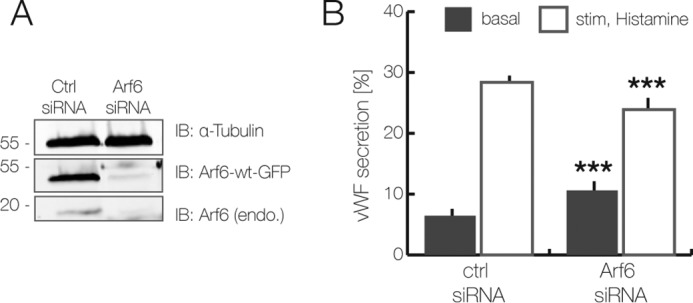
**Arf6 is a positive regulator of acute VWF secretion.** *A* and *B*, siRNA-mediated Arf6 knockdown in HUVEC. Cells were transfected twice for 48 h with an Arf6-specific siRNA or a nonspecific control siRNA. *A*, immunoblot (*IB*) analysis of Arf6-depleted cells, which were co-transfected with wild-type Arf6–GFP. The knockdown efficiency was determined by immunoblotting of total cell lysates using anti-GFP and anti-Arf6-specific antibodies. α-Tubulin served as loading control. *B*, VWF secretion in Arf6-depleted cells. Histamine induced VWF secretion in Arf6-depleted as compared with control siRNA–transfected HUVEC was determined by the ELISA-based secretion assay. Results are expressed as the mean ± S.E. (*error bars*) of 12 independent experiments (***, *p* < 0.001; paired *t* test).

ACAP2 is a known GAP of the small GTPase Arf6, which has mainly been implicated in endocytic processes (27, 28). Hence, we also analyzed whether Arf6 is involved in histamine-evoked WPB exocytosis using the TIRF microscopy-based assay. Whereas overexpression of wild-type Arf6–GFP caused a minor inhibitory effect on rapid WPB disappearance after histamine stimulation, expression of the inactive Arf6(T27N) mutant significantly interfered with Ca^2+^-mediated WPB exocytosis (Fig. 6*B*). To corroborate these findings, we employed the FACS-based VWF secretion assay (see supplemental Fig. S1) and analyzed the effect of Arf6-WT and Arf6(T27N) expression. In line with the TIRF microscopy-based assay, the FACS-based assay also revealed a significant inhibitory effect of Arf6(T27N), whereas overexpression of wild-type Arf6 had no significant consequences (Fig. 6*C*). Next, we also examined whether WPB exocytosis is affected by depletion of Arf6. For this purpose, HUVEC treated with siRNAs targeting Arf6 (see Fig. 7*A* for knockdown efficiency) were subjected to histamine stimulation, and the effect on WPB exocytosis was determined by quantifying the release of VWF into the cell culture supernatant. Evoked VWF release is significantly inhibited in cells depleted of Arf6, whereas the basal VWF secretion is stimulated to some extent (Fig. 7*B*). Together, these results suggest that Arf6 acts as a positive effector of stimulated WPB exocytosis.

**Figure 8.**
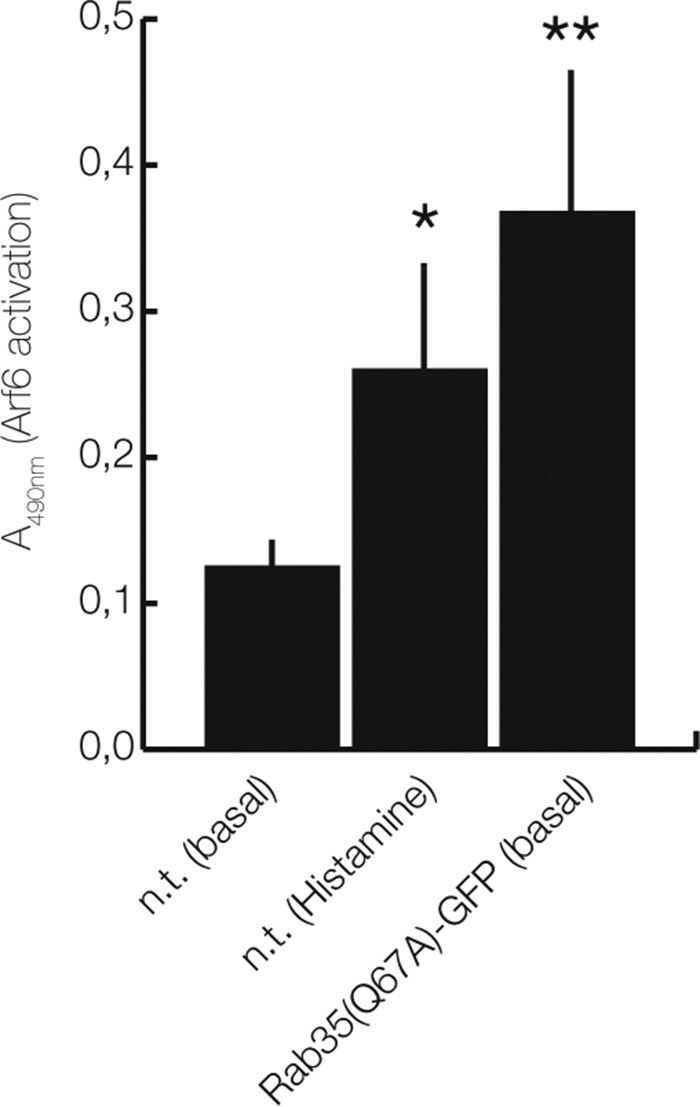
**Rab35(Q67A) expression and histamine stimulation increase Arf6–GTP levels in HUVEC.** HUVEC, either left untreated (non-transfected; *n.t. (basal)*), stimulated with histamine (*n.t. (Histamine)*), or transfected with Rab35(Q67A)-GFP, were lysed, and cell lysates were subjected to an Arf6 activation assay as described under “Experimental procedures.” The levels of Arf6–GTP were quantified in five independent experiments and are expressed as mean ± S.E. (*error bars*) (**, *p* < 0.001; *, *p* < 0.05; unpaired *t* test with Welch's correlation).

Previous reports analyzing different biological processes have shown that Rab35 and Arf6 act in an antagonistic manner in which Rab35 recruits ACAP2 that in turn inhibits Arf6 through its GAP activity (29–33). This appears to be different in the course of histamine-evoked VWF secretion because our experiments indicate that Rab35 and Arf6 both act as positive regulators of this process. To address this unexpected result, we assessed directly whether Rab35 and histamine stimulation can activate Arf6 in HUVEC by quantifying the levels of Arf6–GTP in the accordingly treated cells. Fig. 8 shows that levels of activated, GTP-bound Arf6 are indeed increased in HUVEC expressing Rab35(Q67A). Moreover, when non-transfected HUVEC were treated with the secretagogue histamine, a moderate increase in Arf6–GTP was also observed (Fig. 8). This is in line with our previous findings and suggests that in secretagogue-stimulated HUVEC, both Rab35 and Arf6 support WPB exocytosis.

## Discussion

The exocytosis of WPB is a tightly regulated process enabling endothelial cells to respond to local stimuli by the release of a number of factors initiating coagulation and local inflammation. Regulation is executed at the intracellular level by two second messengers, Ca^2+^ and cAMP, which act through signaling intermediates to activate the exocytosis machinery and trigger the fusion of matured WPB with the plasma membrane and thus the release of WPB cargo. Several of the factors involved in these intracellular steps have been identified, including the Rab GTPases Rab3, Rab15, and Rab27a. Using a comprehensive RabGAP screen, we identify here TBC1D10A as a GAP that, upon overexpression, inhibits histamine-evoked and thus Ca^2+^-dependent WPB exocytosis. Most likely, it acts by inactivating Rab35, because interference with Rab35 function by siRNA-mediated knockdown as well as expression of the dominant-negative Rab35(S22N) mutant also inhibit histamine-stimulated VWF secretion. We cannot exclude the possibility that TBC1D10A also targets other Rab GTPases in the course of WPB exocytosis, in particular, because it also shows GAP activity toward Rab27a (34). However, the function of Rab27a in WPB maturation/exocytosis appears pleiotropic, and it could act as a positive or negative regulator (14, 16). In fact, knockdown of Rab27a has been reported to have either a modest inhibitory or marked stimulatory effect on WPB exocytosis, whereas Rab35 knockdown is clearly inhibitory. Moreover, our experiments show that expression of the GAP-insensitive mutant Rab35(Q67A) rescues the inhibitory effect of TBC1D10A overexpression, in line with Rab35 acting downstream of TBC1D10A.

Rab35 is a novel addition to the list of Rab proteins participating in the maturation and/or evoked exocytosis of WPB. Whereas Rab27a acts inhibitory on exocytosis by linking WPB to the actin cortex and acts stimulatory by recruiting the effector Slp4a (14, 16), the Rab3 isoforms Rab3b and Rab3d are positive regulators, possibly supporting WPB maturation and/or exocytosis (13, 14). Moreover, Rab15 was identified as a positive regulator of WPB exocytosis, which was elicited by ATP/VEGF/basic fibroblast growth factor stimulation, and it appears to act together with Rab27a in this pathway (15). Future experiments will be required to reveal whether these Rabs share common GAPs that regulate their activity and whether Rab35 cooperates with any of the other Rabs in the course of WPB exocytosis.

Rab35 has been shown to regulate endocytic recycling and vesicle trafficking during cytokinesis (for a review, see Ref. 35). Although mainly considered an endocytic Rab, some reports have also linked the protein and its GAPs TBC1D10A to -C to exocytotic processes, such as the secretion of exosomes and the tethering of intracellular vesicles containing apical determinants to the newly forming apical membrane domain in the early steps of epithelial cyst formation (19, 36). In endocytosis, Rab35 is thought to function at two stages (for a review, see Ref. 35). First, through recruiting its effector OCRL, a phosphoinositide phosphatase, Rab35 reduces the level of phosphatidylinositol 4,5-bisphosphate (PI(4,5)P_2_) at newly formed clathrin-coated vesicles and thereby facilitates the uncoating of these early endosomal vesicles (37). Second, by recruiting the effectors MICAL-L1 and ACAP2 to sorting/recycling endosomes, it promotes the generation of tubular recycling endosomes and their exocytosis (29–31, 38, 39). In the course of these events, ACAP2 is thought to function as Arf6GAP, thereby inactivating Arf6 at recycling endosomes. Because Arf6 is a positive regulator of PI(4)P 5-kinase, its inactivation is important to maintain elevated PI(4)P levels at these endosomes, which in turn are required for recruitment and/or stabilization of downstream factors, such as the dynamin-like protein EHD1 (40, 41). Thus, Rab35 and Arf6 act antagonistically in endocytosis and endosomal recycling: Arf6–GTP at the plasma membrane elevates PI(4,5)P_2_ levels via PI(4)P 5-kinase activation and inhibition of Rab35 through binding to its GAPs TBC1D10A to -C (42, 43); Rab35–GTP on endosomes, on the other hand, recruits ACAP2, and the resulting inactivation of Arf6 ensures low PI(4,5)P_2_ levels at endosomes (35). This scenario appears to differ in the control of evoked WPB exocytosis. Here, our data show that both Rab35 and Arf6 support the histamine-triggered secretion of VWF from exocytosing WPB. ACAP2, on the other hand, albeit interacting with Rab35–GTP in endothelial cells, serves as a moderate negative regulator.

How can these, at first sight, contradictory findings be reconciled? Because WPB fuse to the plasma membrane and not to endosomes, different phosphoinositide requirements at the two membranes (plasma membrane *versus* endosomes) could account for different roles of the Rab35–Arf6 axis. Whereas a positive regulatory effect on WPB exocytosis was shown here for the first time for Rab35, Arf6 had been linked to WPB exocytosis before. Huang *et al.* (18) demonstrated that Arf6 is involved in VWF secretion stimulated by Shiga toxin (Stx) subunits 1B and 2B, although they did not observe an effect of Arf6 in histamine-evoked VWF secretion. In Stx-induced WPB exocytosis, Arf6 appears to act in conjunction with phospholipase D1 (PLD1), the enzyme catalyzing phospholipid conversion from phosphatidylcholine to phosphatidic acid (PA); PLD1 is activated by Arf6, and PLD1 knockdown inhibits Stx-triggered VWF secretion. PLD1 also participates in histamine-evoked VWF secretion. It is activated by histamine treatment, and PA elevation is observed in the plasma membrane of histamine-stimulated endothelial cells (11). Similar to Arf6, PA can also activate PI(4)P 5-kinase, and the product of PI(4)P 5-kinase catalysis, PI(4,5)P_2_, can in turn stimulate PLD1 (28, 44). Thus, it seems that Arf6, PA, and PI(4,5)P_2_ act in a positive feedback scenario to stimulate evoked exocytosis of WPB in endothelial cells. Provided that Rab35 and Arf6 do not counteract but both support evoked WPB exocytosis, a direct link between the two GTPases via the Arf6GAP ACAP2 can be explained by two possible mechanisms. First, Rab35–GTP could bind ACAP2 and thereby inactivate this GAP at the endothelial plasma membrane, possibly in conjunction with other effectors. This would result in enhanced Arf6–GTP levels at the plasma membrane, which in turn stimulate PLD1 and thereby facilitate WPB exocytosis. Second, Rab35–GTP could sequester ACAP2 to intracellular organelles, including most likely recycling endosomes and some internal WPB (see Fig. 3), and this could increase the level of Arf6–GTP at the plasma membrane where WPB fusion occurs. Our first results (Fig. 8) indeed indicate that Arf6–GTP levels are increased in HUVEC expressing Rab35(Q67A) or stimulated with histamine. Future experiments will be required to reveal whether a Rab35-mediated activation of Arf6 occurs through sequestering/inactivation of ACAP2 or whether other mechanisms are involved.

Although our results suggest that Rab35 acts upstream of Arf6 to directly support WPB exocytosis at the level of the plasma membrane, other modes of action are also compatible with our data. Based on the Rab35 activity in the endosomal system, it remains possible that it acts indirectly by enhancing the recycling of components of the WPB fusion machinery (*e.g.* plasma membrane-resident SNAREs) back to the plasma membrane. Rab35 could also be involved in the re-endocytosis of membrane material after WPB fusion and/or the re-internalization of WPB membrane that has not fully flattened into the plasma membrane after WPB fusion. A block of such uptake could possibly feed back onto the secretion machinery, resulting in the reduction of exocytotic fusion events. In such cases, Rab35 most likely operates independently of Arf6 because their antagonistic actions in endocytosis have been shown in several system (35). Future work will be required to reveal the exact mechanism underlying the function of Rab35 in WPB exocytosis, for example by visualizing a possible enrichment of Rab35 at WPB fusion sites by high-resolution live cell microscopy.

## Experimental procedures

### Antibodies, plasmids, and other reagents

The following primary antibodies were used: rabbit anti-VWF (Dako), mouse anti-VWF (clone F8/86, Dako), rabbit anti-GFP (Invitrogen), rabbit anti-GFP (Novus Biologicals), mouse anti-α-actin (clone C4, Millipore), mouse anti-β-actin (clone AC-15, Sigma-Aldrich), mouse anti-GST (clone B14, Santa Cruz Biotechnology, Inc.), mouse anti-Myc (clone 9E10, Invitrogen), rabbit anti-c-Myc (clone A14, Santa Cruz Biotechnology), rabbit anti-Rab35 (Proteintech), rabbit anti-TBC1D10A (Abcam), rabbit anti-ACAP2 (Sigma-Aldrich), rabbit anti-Arf6 (D12G6, Cell Signaling), rabbit anti-VWF-DyLight® 650 (generated with the DyLight® antibody labeling kit from Thermo Scientific by coupling the dye to rabbit anti-VWF from Dako), rabbit anti-tubulin (clone B-5-2-2, Sigma), sheep anti-VWF-FITC (Abcam), and mouse primary antibody isotype control (Invitrogen). Secondary antibodies for Western blots were purchased from LI-COR, coupled either to IRDye® 800CW or IRDye® 680RD infrared dye. The secondary antibody for microscopy was anti-mouse Alexa Fluor 568.

Transient transfections were carried out with the following expression plasmids: C1-pEGFP (Clontech, Mountain View, CA); EGFP-tagged Rab GAPs and their catalytically inactive mutants (45); VWF-GFP (kindly provided by Tom Carter (St. George's, University of London)); ACAP2, Rab35, Rab35(S22N), and Rab35(Q67A) as GFP-tagged variants (generated by site-directed mutagenesis and cloning into pEGFP); and Arf6 and Arf6(T22N), which were also employed as GFP-tagged versions (kindly provided by Andreas Püschel (Institute for Molecular Cell Biology, Westfälische Wilhelms-Universität Münster)). A P-selectin luminal domain construct coupled to mRFP, herein referred to as Psellum–RFP, was kindly provided by Daniel Cutler (MRC Laboratory for Molecular Cell Biology, University College London). The different siRNAs employed were purchased from Sigma-Aldrich, and the nonspecific AllStars negative control siRNA was from Qiagen (Valencia, CA).

### Cell culture and transfection

HUVEC were isolated from umbilical cord veins as described previously (46). They were maintained in endothelial cell growth medium (ECGM2 (Promocell) supplemented with 20 μg/ml gentamycin, 15 μg/ml amphotericin B) until passage 1 and then cultivated in mixed ECGM2 (mixed 1:2 with M199 (Biochrom), supplemented with 10% FCS, 20 μg/ml gentamycin, 15 μg/ml amphotericin B, and 100 international units (IU) heparin). Cells were used at passages 3–4 and 80–90% confluence and transfected with plasmids or siRNAs using the Amaxa Nucleofection Technology (HUVEC Nucleofector Kit-OLD, Lonza) according to the manufacturer's instructions (13). For rescue experiments, cells were transfected with siRNAs, cultivated for 48 h, and then transfected again with the specific siRNA, but this time together with the particular siRNA-insensitive GFP expression construct. Knockdown efficiency was confirmed by immunoblotting. Therefore, HUVEC grown in 100-mm dishes were trypsinized, washed twice with PBS, resuspended in 100 μl of lysis buffer (20 mm HEPES (pH 7.4), 150 mm NaCl, 0.5% Triton X-100, 1.5 mm PMSF, 1× protease inhibitor), sonicated, and incubated on ice for 15 min. Following centrifugation (10 min, 1250 × *g*, 4 °C), the postnuclear supernatant was mixed with SDS sample buffer, separated in either 12 or 15% SDS-polyacrylamide (PAA) gels, and subjected to immunoblotting using specific first and infrared-labeled secondary antibodies (LI-COR). Bands were visualized using an Odyssey imager.

### FACS-based secretion assay

To quantify the effect of overexpression of a RabGAP library on stimulated WPB exocytosis, a FACS-based VWF secretion assay was developed. Therefore, HUVEC were transfected with the individual GFP-tagged RabGAPs using Amaxa nucleofection technology (HUVEC nucleofector kit-OLD, Lonza) according to the manufacturer's instructions. 24 h post-transfection (hpt), one part of the transfected cells was stimulated for 20 min with 100 μm histamine (Sigma) in basal medium (1% (w/v) BSA in M199, supplemented with rabbit anti-VWF-DyLight^TM^ 650 antibodies), whereas the other part was treated with basal medium alone. Cells were washed with warm PBS and detached from the cell culture dish using Accutase (PAA). Subsequently, the cells were centrifuged (4 min, 150 × *g*) and suspended in PBS before paraformaldehyde fixation. Cells were spun down again (4 min, 150 × *g*) and washed in PBS, and anti-VWF-positive cell populations were identified directly by flow cytometry in a FACSCalibur^TM^ cell analyzer (BD Biosciences) using CellQuest Pro version 4.0.2.

### ELISA-based VWF secretion assay and Arf6 G-LISA activation assay

The amount of VWF secreted from a confluent HUVEC culture was quantified by ELISA as described before (17, 47). HUVEC were stimulated for 20 min with 100 μm histamine (Sigma-Aldrich), and three successive triplicate samples were analyzed per condition (supernatants of non-stimulated cells, of stimulated cells, and of the residual total lysates). The values obtained were expressed as a percentage of total VWF content.

A G-LISA Arf6 activation assay (Biochem kit, Cytoskeleton) was used to measure Arf6–GTP levels in HUVEC. Cells expressing Rab35(Q67A) or cells stimulated for 20 min with 100 μm histamine were lysed, and the lysates were applied to wells of a 96-well Arf6–GTP affinity plate and incubated for 30 min at 4 °C. Following washing, active Arf6–GTP bound to the wells was detected with a specific monoclonal anti-Arf6 antibody and HRP-labeled secondary antibodies according to the manufacturer's instructions. The degree of Arf6 activation was determined by comparing the level of Arf6–GTP in mock-transfected, non-stimulated cells with the Arf6–GTP level in Rab35(Q67A)-expressing or histamine-stimulated HUVEC.

### GST pulldown and co-immunoprecipitation

To identify Rab35 effectors in an affinity chromatography approach, HUVEC lysates from 15 100-mm culture dishes were incubated with glutathione-Sepharose (GSH) beads (GE Healthcare) coupled to Rab35–GST or GST, respectively, in the presence of 100 μm GTPγS for 2 h at 4 °C. After several washing steps, the samples were treated with 5 mm GDP (supplemented with 20 mm EDTA, 1 mm DTT, 1.5 m NaCl, and 20 mm Hepes), and eluted proteins were separated in 10% SDS-PAA gels and stained using the ProteoSilver^TM^ Plus silver stain kit (Sigma-Aldrich) according to manufacturer's instructions.

To verify the interaction of Rab35 with ACAP2, GSH beads coupled to 3 μg of Rab35-WT–GST, Rab35(S22N)–GST, Rab35(Q67A)–GST, or GST alone, respectively, were incubated for 3–4 h at 4 °C with HEK-293T cell lysates containing ACAP2–GFP. After several washing steps, bound proteins were eluted with SDS sample buffer, separated in 12% SDS-PAA gels, and immunoblotted using specific first and infrared-labeled secondary antibodies (LI-COR). The bands were visualized using an Odyssey imager.

For co-IPs, HEK-293T cells were transfected with Myc-tagged Rab35 derivatives together with catalytically inactive RA mutants of the different Rab GAPs or ACAP2–GFP, respectively. In the case of ACAP2 co-IPs, cells were lysed in an IP buffer containing 25 mm Tris/HCl (pH 7.4), 150 mm NaCl, 1 mm EDTA, 1% Nonidet P-40, and 5% glycerol. In the case of TBC1D10 co-IPs, cells were incubated with 10 mm NaF and 100 μm AlCl_3_ for 30 min at 37 °C to activate the GTPase and then lysed in RabGAP buffer containing 1% Brij® 97, 150 mm NaCl, 10 mm Tris/HCl (pH 7.5), 1 mm CaCl_2_, 1 mm MgCl_2_, 1% Triton X-100, 0.02% NaN_3_, 10 mm NaF, 100 μm AlCl_3_, 0.1 3,3′-dithiobis(sulfosuccinimidyl proprionate) (DTSSP), and a complete protease inhibitor mixture (Roche Applied Science). Cell lysates were subjected to a preclearing step with non-conjugated Dynabeads (Invitrogen) to remove proteins binding nonspecifically to the beads. Thereafter, the immunoprecipitation was carried out with Dynabeads coupled to the primary antibody (2 h, 4 °C). Subsequently, the beads were washed four times with IP buffer or RabGAP buffer, respectively, and precipitated proteins were eluted in SDS sample buffer, separated by SDS-PAGE, and subjected to Western blot analysis.

### Immunofluorescence, microscopy, and image analysis

Cells were grown on collagen-coated coverslips or chambered slides (Nunc Labtek) for 48 h after transfection, if not stated otherwise. For analysis of fixed cells, samples were treated with 4% paraformaldehyde (10 min, room temperature), permeabilized with 0.2% Triton X-100 (2 min, room temperature), labeled with fluorescent antibodies, and mounted in Mowiol. Live cells were imaged in endothelial cell growth medium supplemented with 25 mm HEPES (pH 7.4) at 37 °C. Confocal imaging employed a Zeiss LSM780 confocal microscope and a Plan-Apochromat ×63/1.4 numeric aperture oil immersion objective. Confocal videos were recorded at different speeds. TIRF imaging was performed with an Olympus IX71 TIRF microscope customized to include a heated incubation chamber, an objective-type TIRFM setup (TILL Photonics), a monochromator for epifluorescence excitation, and a controller allowing hardware-controlled fast switching between TIRF and epifluorescence (TILL Photonics). Images were acquired using a QE charge-coupled device camera (TILL Photonics) and MetaMorph Software (Molecular Devices). The total internal reflection angle was adjusted manually for every experiment. TIRF time-lapse movies were recorded with 2 frames/s.

Image analysis was performed in ImageJ. Objects in the TIRF field positive for Psellum–mRFP as a WPB marker were quantified with an image segmentation algorithm named MorphoQuant (48). This algorithm allows the detection of locally bright and large objects of arbitrary shape over time by using a top hat filter with a circular structural element whose radius, *r*_1_, was defined in such a way as to include a typical WPB (*r*_1_ = 15 px; 1 px ≈ 133 nm). To quantify the Psellum-mRFP–positive objects per cell, images in the RFP detection channel were masked as follows: one mask for each cell containing Psellum-mRFP–positive objects and another, so-called background mask that was placed outside the cellular masks covering no signal. The second mask served as a reference area to normalize fluorescence values over time. The distinction between foreground and background was further improved by subsequent filtering, whereby the relative fluorescence intensity was defined by an adjustable threshold *t*_1_ (*t*_1_ = 8 arbitrary units (a.u.)). Data were prepared in Excel to illustrate the number and fluorescence of Psellum-mRFP–positive objects over time.

### Statistical analysis

The significance of data were evaluated with the statistical program GraphPad Prism version 6 using either a paired *t* test or one-way ANOVA with a subsequent Tukey test.

## Author contributions

A. B. and A. G. performed experiments, analyzed data, and wrote the manuscript; F. B. generated tools and wrote the manuscript; V. G. analyzed data and wrote the manuscript.

## Supplementary Material

Supplemental Data

## Footnotes

3 The abbreviations used are:

VWF: von Willebrand factor
WPB: Weibel–Palade body/bodies
HUVEC: human umbilical vein endothelial cell(s)
mRFP: monomeric RFP
RA: arginine to alanine
IP: immunoprecipitation
TIRF: total internal reflection fluorescence
PI(4,5)P_2_: phosphatidylinositol 4,5-bisphosphate
PI(4)P: phosphatidylinositol 4-phosphate
PA: phosphatidic acid
EGFP: enhanced GFP
PAA: polyacrylamide
hpt: hours post-transfection
GTPγS: guanosine 5′-3-*O*-(thio)triphosphate
GAP: GTPase-activating protein
ANOVA: analysis of variance
G-LISA: GTPase-linked immunosorbent assay.
