# Supplementary material for: Rab35 protein regulates evoked exocytosis of endothelial Weibel–Palade bodies
Source: J Biol Chem. 2017 May 31;292(28):11631–40. doi: 10.1074/jbc.M116.773333 (PMC5512060; doi:10.1074/jbc.M116.773333)
Supplement: Supplemental Data [file supp_292_28_11631__index.html]

Rab35 Regulates Evoked Exocytosis of Endothelial Weibel-Palade Bodies — Rab35 protein regulates evoked exocytosis of endothelial Weibel–Palade bodies — Rab35 in Weibel–Palade body exocytosis — Supplemental Data 

# Rab35 protein regulates evoked exocytosis of endothelial Weibel–Palade bodies

## Supplemental Data

- Supplemental figures (.pdf, 3.2 MB) - Revised supplemental figures
