# Supplementary material for: Rab35 protein regulates evoked exocytosis of endothelial Weibel–Palade bodies
Source: J Biol Chem. 2017 May 31;292(28):11631–40. doi: 10.1074/jbc.M116.773333 (PMC5512060; doi:10.1074/jbc.M116.773333)
Supplement: Supplemental Data [file 10.1074_M116.773333_jbc.M116.773333-1.pdf]

Supplemental figure legends

S1. Immunological quantification of exocytosed VWF at the cell surface.

(A) Schematic representation of histamine-induced capture of anti-vWF-antibodies. Histamine triggers the Ca^{2+}-mediated exocytosis of WPBs in endothelial cells. Extracellular anti-VWF antibodies bind the released VWF at the cell surface and capture it into VWF-anti-VWF antibody complexes. (B) Distribution of internal and external VWF. HUVEC were transfected with VWF-RFP and grown on coverslips for 24 h. Cells were then either kept in basal medium or stimulated with histamine for 20 min. In both cases the extracellular medium contained FITC-labelled anti-VWF antibodies to capture released VWF at the cell surface. Subsequently, cells were fixed and analyzed by confocal microscopy. Note that histamine stimulated cells show a significant increase of surface complexes containing the FITC labelled anti-VWF antibodies. Scale bar: 10 µm. (C) Histogram depicting the fluorescence intensity of FITC-labelled anti-VWF antibodies at the cell surface of non-stimulated (basal) or histamine-stimulated HUVEC, as obtained by flow cytometry.

S2. Depletion of TBC1D10A increases the histamine-evoked secretion of VWF.

(A) TBC1D10A and TBC1D10B knockdown in HUVEC. Cells were transfected twice for 48 h with siRNAs specific for TBC1D10A and TBC1D10B, respectively, as well as with an unspecific control siRNA. Shown are Immunoblot analyses of TBC1D10A/B depleted cells, which were co-transfected with TBC1D10A/B-GFP expression constructs. Blots were either probed with anti-GFP antibodies (upper panels) or with a TBC1D10A-specific antibody (lower panel, left) which recognizes the endogenous (arrow) as well as the GFP-tagged protein (asterisk). An antibody specifically recognizing the endogenous TBC1D10B was unfortunately not available. α-actin was used as loading control. (B) VWF secretion levels in TBC1D10A/B depleted cells. HUVEC were treated with siRNAs as described in (A) and stimulated with histamine for 20 min. Acute VWF secretion was then quantified by ELISA. Results are expressed as the mean ±SEM of eight independent experiments (*** $p<0.0001$; paired t-test).

S3. Subcellular distribution of TBC1D10A and TBC1D10B.

HUVEC were grown on coverslips and transfected with plasmids encoding VWF-RFP and TBC1D10A-GFP or TBC1D10B-GFP, respectively. Cells were fixed 24 hpt and analysed by confocal microscopy. The representative images display maximum-intensity projections. Scale bar: 10 µm.

S4. Live cell imaging of Rab35 in histamine stimulated HUVEC.

HUVEC expressing Rab35-wt-GFP and VWF-RFP as WPB marker were stimulated with histamine and subjected to confocal live cell microcopy. Shown are stills of a movie focusing on three WPB. Stimulation occurred at t = 0 sec. Note that the WPB in the center has fused with the plasma membrane at t = 16 sec (fusion is characterized by change of the elongated intracellular VWF-RFP signal into a diffuse round appearance), whereas the other two WPB did not fuse. Note also that Rab35-GFP accumulates at the site of WPB fusion. Scale bar: 10 µm.

S5. Identification of Rab35 effectors via affinity chromatography.

A GST pulldown from HUVEC lysates with Rab35-wt-GST and GST was performed according to Material and methods. Bound proteins were eluted, separated by SDS-PAGE and visualized by silver staining. The bands indicated were excised and subjected to mass spectrometry analysis. Proteins were identified as follows: (1) unknown, (2) ACAP2, (3) Chaperon DnaK (*E. coli*), (4) Hsc71 (isoform 1), (5) PRO2619, (6) Keratin 1 and 10, (7) not identified, (8) Keratin1 and 10.

S6. Live cell imaging of ACAP2 in histamine stimulated HUVEC.

HUVEC expressing ACAP2-GFP and VWF-RFP as WPB marker were stimulated with histamine and subjected to confocal live cell microcopy. Shown are stills of a movie focusing on one WPB that fuses with the plasma membrane between t = 0 sec (time of stimulation) and t = 10 sec. Note that the general ACAP2 distribution is similar to that seen in non-stimulated HUVEC and does not change upon WPB fusion (Fig. 5C). Scale bar: 10 µm.

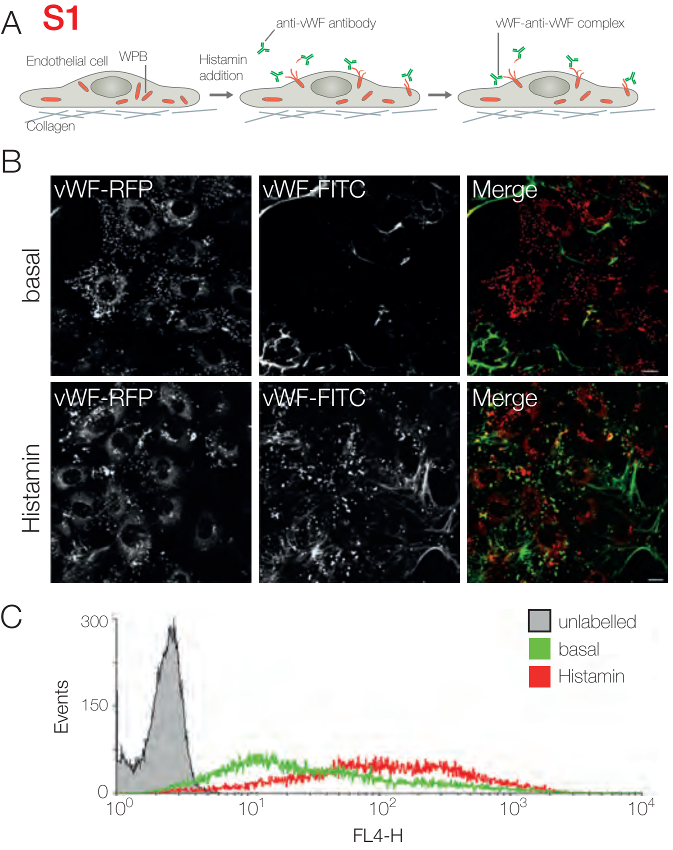

A
S1
Endothelial cell
WPB
Collagen
Histamin addition
anti-vWF antibody
vWF-anti-vWF complex
B
basal
Histamin
vWF-RFP
vWF-FITC
Merge
C
unlabelled
basal
Histamin
Events
300
150
0
FL4-H

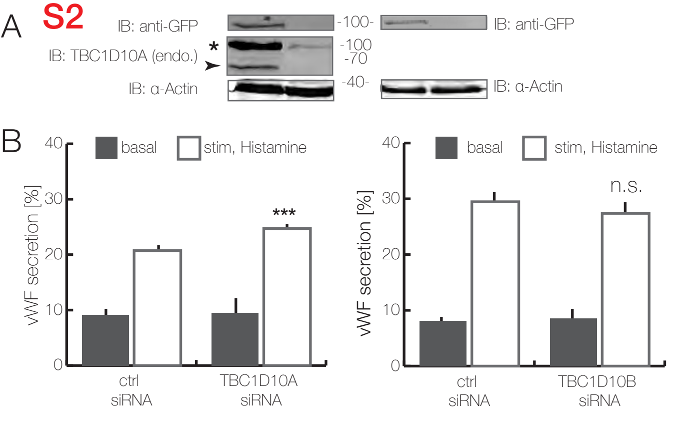
S2
A
IB: anti-GFP
-100-
IB: anti-GFP
IB: TBC1D10A (endo.)
*
-100
-70
IB: α-Actin
-40-
IB: α-Actin
B
basal
stim, Histamine
vWF secretion [%]
40
30
20
10
0
***
ctrl siRNA
TBC1D10A siRNA
basal
stim, Histamine
n.s.
TBC1D10B siRNA

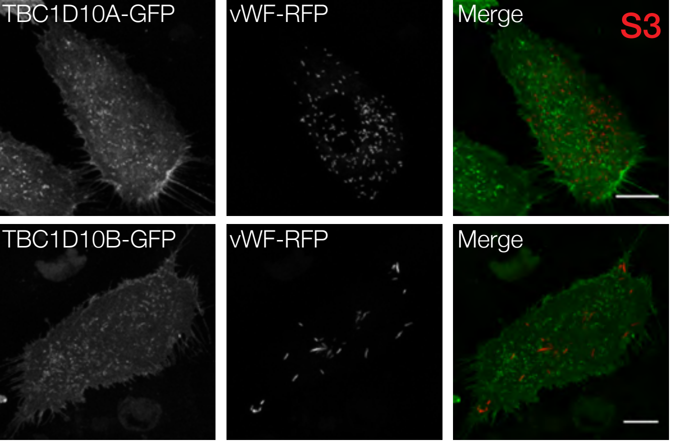
TBC1D10A-GFP
vWF-RFP
Merge
S3
TBC1D10B-GFP
vWF-RFP
Merge

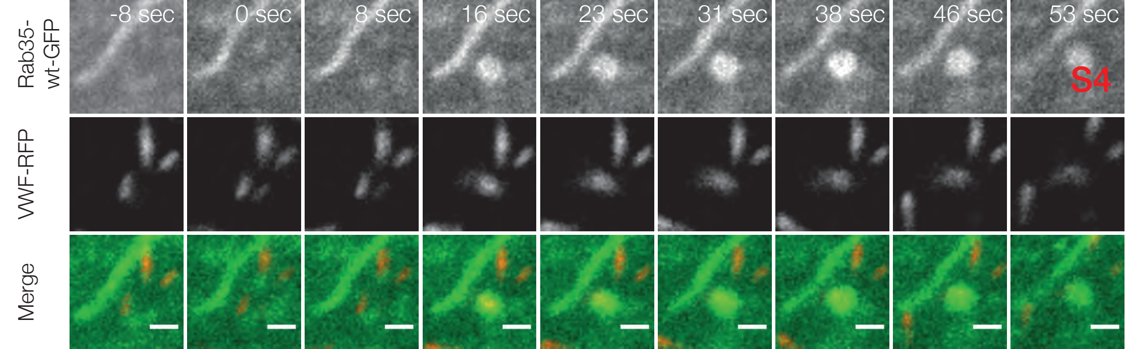

Rab35-wt-GFP
VWF-RFP
Merge
-8 sec
0 sec
8 sec
16 sec
23 sec
31 sec
38 sec
46 sec
53 sec
S4

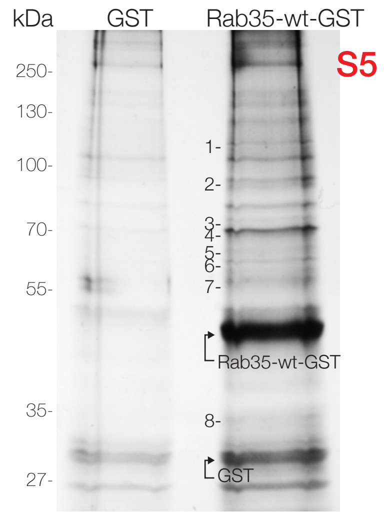
kDa
GST
Rab35-wt-GST
S5
250-
130-
100-
70-
55-
35-
27-
1-
2-
3-
4-
5-
6-
7-
Rab35-wt-GST
8-
GST

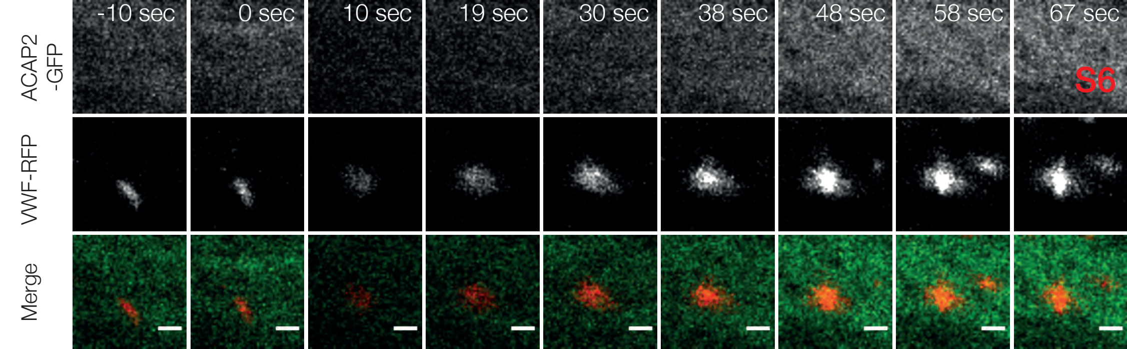
-10 sec
0 sec
10 sec
19 sec
30 sec
38 sec
48 sec
58 sec
67 sec
ACAP2
-GFP
VWF-RFP
Merge
S6
